# A preclinical evaluation of the MEK inhibitor refametinib in HER2-positive breast cancer cell lines including those with acquired resistance to trastuzumab or lapatinib

**DOI:** 10.18632/oncotarget.19461

**Published:** 2017-07-22

**Authors:** John O’Shea, Mattia Cremona, Clare Morgan, Malgorzata Milewska, Frankie Holmes, Virginia Espina, Lance Liotta, Joyce O’Shaughnessy, Sinead Toomey, Stephen F. Madden, Aoife Carr, Naomi Elster, Bryan T. Hennessy, Alex J. Eustace

**Affiliations:** ^1^ Department of Medical Oncology, Royal College of Surgeons in Ireland, Beaumont Hospital, Beaumont, Ireland; ^2^ Texas Oncology-Memorial Hermann Memorial City, US Oncology Research, Houston, TX, USA; ^3^ George Mason University, Manassas, VA, USA; ^4^ Baylor-Sammons Cancer Center, Texas Oncology, US Oncology, Dallas, TX, USA; ^5^ Data Science Centre, Royal College of Surgeons in Ireland, Dublin, Ireland

**Keywords:** MEK inhibitor, HER2-positive breast cancer, acquired resistance to HER2-targeted therapies, reverse phase protein array

## Abstract

**Purpose:**

The MEK/MAPK pathway is commonly activated in HER2-positive breast cancer, but little investigation of targeting this pathway has been undertaken. Here we present the results of an *in vitro* preclinical evaluation of refametinib, an allosteric MEK1/2 inhibitor, in HER2-positive breast cancer cell lines including models of acquired resistance to trastuzumab or lapatinib.

**Methods:**

A panel of HER2-positive breast cancer cells were profiled for mutational status and also for anti-proliferative response to refametinib alone and in combination with the PI3K inhibitor (PI3Ki) copanlisib and the HER2-targeted therapies trastuzumab and lapatinib. Reverse phase protein array (RPPA) was used to determine the effect of refametinib alone and in combination with PI3Ki and HER2-inhibitors on expression and phosphorylation of proteins in the PI3K/AKT and MEK/MAPK pathways. We validated our proteomic *in vitro* findings by utilising RPPA analysis of patients who received either trastuzumab, lapatinib or the combination of both drugs in the NCT00524303/LPT109096 clinical trial.

**Results:**

Refametinib has anti-proliferative effects when used alone in 2/3 parental HER2-positive breast cancer cell lines (HCC1954, BT474), along with 3 models of these 2 cell lines with acquired trastuzumab or lapatinib resistance (6 cell lines tested). Refametinib treatment led to complete inhibition of MAPK signalling. In HCC1954, the most refametinib-sensitive cell line (IC_50_ **=** 397 nM), lapatinib treatment inhibits phosphorylation of MEK and MAPK but activates AKT phosphorylation, in contrast to the other 2 parental cell lines tested (BT474-P, SKBR3-P), suggesting that HER2 may directly activate MEK/MAPK and not PI3K/AKT in HCC1954 cells but not in the other 2 cell lines, perhaps explaining the refametinib-sensitivity of this cell line. Using RPPA data from patients who received either trastuzumab, lapatinib or the combination of both drugs together with chemotherapy in the NCT00524303 clinical trial, we found that 18% (n=38) of tumours had decreased MAPK and increased AKT phosphorylation 14 days after treatment with HER2-targeted therapies. The combination of MEK inhibition (MEKi) with refametinib and copanlisib led to synergistic inhibition of growth in 4/6 cell lines tested (CI @ED_75_ = 0.39-0.75), whilst the combinations of lapatinib and refametinib led to synergistic inhibition of growth in 3/6 cell lines (CI @ED_75_ = 0.39-0.80).

**Conclusion:**

Refametinib alone or in combination with copanlisib or lapatinib could represent an improved treatment strategy for some patients with HER2-positive breast cancer, and should be considered for clinical trial evaluation. The direct down-regulation of MEK/MAPK but not AKT signalling by HER2 inhibition (e.g. by lapatinib or trastuzumab), which we demonstrate occurs in 18% of HER2-positive breast cancers may serve as a potential biomarker of responsiveness to the MEK inhibitor refametinib.

## INTRODUCTION

Breast cancer is the most prevalent form of malignancy in females [1]. Between 20% to 30% of breast cancers over-express the human epidermal growth factor receptor 2 (HER2)/avian erythroblastosis oncogene B2 (ERBB2) protein on their cell surface [2], and over-expression of HER2 is strongly linked to worse clinical prognosis [3]. Trastuzumab (Herceptin), a monoclonal antibody that interferes with HER2 signalling, has improved clinical outcome in patients with HER2-positive breast cancers [4, 5], however it is apparent that up to 66% of patients exhibit resistance to trastuzumab monotherapy [4, 5].

Lapatinib (Tykerb), a small molecule tyrosine kinase inhibitor which targets both epidermal growth factor receptor (EGFR) and HER2, is effective in the treatment of some trastuzumab-resistant HER2-positive breast cancers *in vitro* and *in vivo* [6–8]. However, not all HER2-positive breast cancer cells respond to lapatinib [9]. Mechanisms of resistance to lapatinib have been described, including gene mutations in effector proteins which allow for activation of intercellular signalling cascades such as the phosphatidylinositol 3’ kinase (PI3K)-AKT (PI3K/AKT) and Raf-MEK-ERK mitogen-activated protein kinase (MEK/MAPK) pathways [10].

Previous studies have shown that cell lines overexpressing HER2 and HER2-positive breast cancer have an activated PI3K/AKT pathway [10], however HER2 activation also activates the MEK/MAPK pathway [11]. In this pathway the ERBB receptor activates membrane bound RAS, allowing RAS to bind to multiple effector proteins, most notably, RAF proteins. RAF proteins activate MEK1 by phosphorylation, which then activates the extracellular signal-related kinases, ERK-1 and ERK-2, resulting in increased cell proliferation, differentiation and reduced apoptosis.

Many clinical and preclinical studies are currently investigating the importance of targeting PI3K in HER2-positive breast cancer, however the MEK/MAPK pathway has also been recently established as a potential target for therapy in oncology patients [12]. Interestingly studies by Cheng *et al* have found that PIK3CA mutated HER2-positive breast cancer tumours escape PIK3CA dependence by activating MAPK/MEK signalling pathways [13]. In fact current trials of the MEK inhibitor trametinib in triple negative breast cancer are underway (NCT01964924). However to date no-one has studied the role of MEK inhibition in HER2-positive breast cancer. We propose to investigate the preclinical efficacy of BAY86-9766 (refametinib), an allosteric MEK inhibitor, in models of HER2-positive breast cancer (parental cells (-P)) and in matched models with acquired resistance to trastuzumab (-T and -Res) and lapatinib (-L).

## RESULTS

### Refametinib sensitivity and proteomic profiles of SKBR3, HCC1954 and BT474 cells

As previously shown by us mutations in the PIK3CA gene were identified in BT474 (K111N) and HCC1954 (H1047R) [22]. The mutational status of PI3K did not change between parental cell lines and models of acquired resistance to trastuzumab or lapatinib (Table 1).

**Table 1:** IC_50_ values for refametinib, copanlisib, lapatinib and the effect of trastuzumab on growth inhibition in a panel of HER2-positive breast cancer cell lines including parental cells (-P) and matched models of acquired trastuzumab (-T and -Res) and lapatinib (-L) resistance

Cell Line	Acquired resistance	PIK3CA mutation Status	p53 mutation Status	BAY86-9766 refametinib (MEKi) (nM)	BAY80-6946 copanlisib (PI3Ki) (nM)	Lapatinib (nM)	Trastuzumab (% inhibition @10μg/ml)
SKBR3-P	N/A	WT	R175H	>4000	13.2 ± 3.4	57.3 ± 6.6	37.6 ± 6.4
SKBR3-L	L	WT	N/A	>4000	45.2 ± 4.3	1237.7 ± 212.7	15.9 ± 8.2
SKBR3-T	T	WT	N/A	>4000	12.4 ± 3.5	79.3 ± 12.5	10.6 ± 5.3
HCC1954-P	N/A	H1047R	Y163C	357.3 ± 87.8	9.0 ± 0.4	581.0 ± 84.5	-10.0 ± 19.0
HCC1954-L	L	H1047R	N/A	713.7 ± 160.2	10.3 ± 0.9	3602.0 ± 311.3	-2.0 ± 14.0
BT474-P	N/A	K111N	E285K	1245.3 ± 152.0	1.8 ± 0.6	14.3 ± 5.0	39.8 ± 4.9
BT474-RES	T	K111N	N/A	1379.3 ± 190.5	4.1 ± 0.9	223.7 ± 48.5	8.21 ± 5.2

PIK3CA and p53 mutational analysis acquired from the Broad Institute CCLE website (http://www.broadinstitute.org/ccle/home). PIK3CA and p53 mutational analysis acquired from the Broad Institute CCLE website (http://www.broadinstitute.org/ccle/home). Standard deviations are representative of triplicate independent experiments. N/A indicates parental cell lines which do not have acquired resistance or cell lines in which mutation status has not been determined. WT = wild-type.

The MEK inhibitor BAY 86-9766/RDEA0119 (refametinib) achieved an IC_50_ in the parental HCC1954-P (357.33 ± 87.75 nM) and BT474-P (1245.33 ± 151.95 nM) cells but failed to achieve an IC_50_ at 4μM in SKBR3 cells. As a point of reference colorectal cell lines with BRAF mutations, which would be regarded as sensitive in general have IC_50_s for refametinib ranging from 50nM to >1000 nM [23, 24], whilst the triple negative breast cancer cell line MDAMB231 has an IC_50_ of less than 100nM [25]. We observed a similar pattern of cell line sensitivity to the alternative MEKi GDC-0973, with HCC1954-P cells being the most sensitive cell lines tested with an IC_50_ of 1563 ± 224 nM. In SKBR3-P and BT474-P cells GCD-0973 failed to achieve an IC_50_ but instead inhibited growth in the SKBR3-P and BT474-P cells by 35.6 ± 8.3% and 25.2 ± 4.3% respectively. Furthermore we looked at *in-vitro* sensitivity to the MEK inhibitors which are currently under clinical evaluation in triple negative breast cancer and found that 10/15 triple negative cells analysed were sensitive to MEK inhibitors (PD-0325901 and trametinib) we also observed that 12/17 HER2-positve breast cancer cells lines were also sensitive (Supplementary Table 3).

We also tested refametinib in models of acquired resistance to either lapatinib or trastuzumab which had been established by continual exposure to the relevant drug for at least 6 months. Both SKBR3-L and HCC1954-L cell lines resistance to lapatinib is reported to be associated with reduced phosphorylation of eukaryotic elongation factor 2 [15]. Our RPPA analysis found that HCC1954-L cells have significantly elevated p-MAPK (Y202/T204) (p=0.008) and p-MEK (S217/221) (p=0.002) relative to matched parental controls, whilst SKBR3-L cells have reduced p-AKT (T308) (p=0.05) and p-HER2 (Y1248) (p=0.01) levels relative to matched parental cell lines (Supplementary Figure 1). Refametinib was significantly less effective in the lapatinib resistant HCC1954-L cells relative to matched parental cells (IC_50_ = 713.66 ± 160.23 nM, p = 0.027), however in the BT474-RES trastuzumab resistant cells the IC_50_ was unchanged relative to matched parental cells. As previously reported [22] the HER2-positive breast cancer cell lines HCC1954, BT474 and SKBR3 are sensitive to copanlisib and lapatinib at nM concentrations (Table 1), whilst only BT474 and SKBR3 cells have sensitivity *in vitro* to trastuzumab.

**Figure 1:**
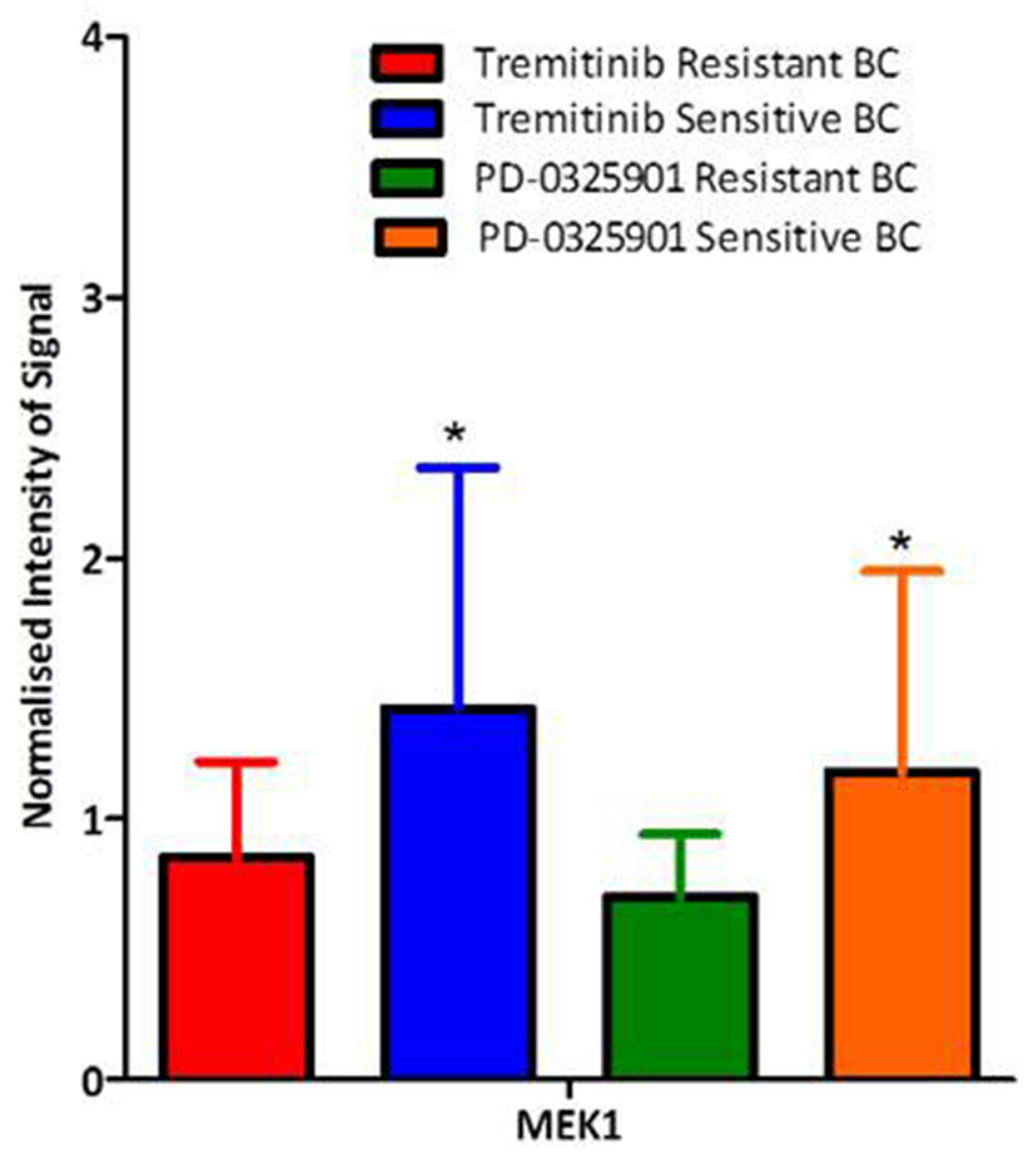
Differential expression of MEK1 as determined by RPPA in a panel of breast cancer cell lines (n=28) dependant on their sensitivity to the MEK inhibitors tremitinib and PD-0325901 Standard deviations are calculated from analysis of MEK expression in the sensitive (n=15) versus the resistant (n=12) cell lines analysed on the same RPPA slide. ‘^*^’ indicates a significant p value of <0.05 as determined by the students t-test.

HER2-positive BT474-P and HCC1954-P breast cancer cells thus have some innate sensitivity to refametinib [23, 24] while SKRB3-P cells are *de novo* resistant. Using the GDSC database we determined the IC_50_ of a panel of breast cancer cell lines to both trametinib and PD-0325901. Using RPPA analysis by Daemen *et al* [26], we then determined the basal proteomic signature of each cell lines across 70 antibodies representing multiple nodes of the PI3/AKT and MAPK/ERK signalling pathway (Figure 1). Our results indicate that cells which had higher basal levels of MEK1 expression were significantly more likely to be sensitive to MEK inhibitors.

**Figure 2:**
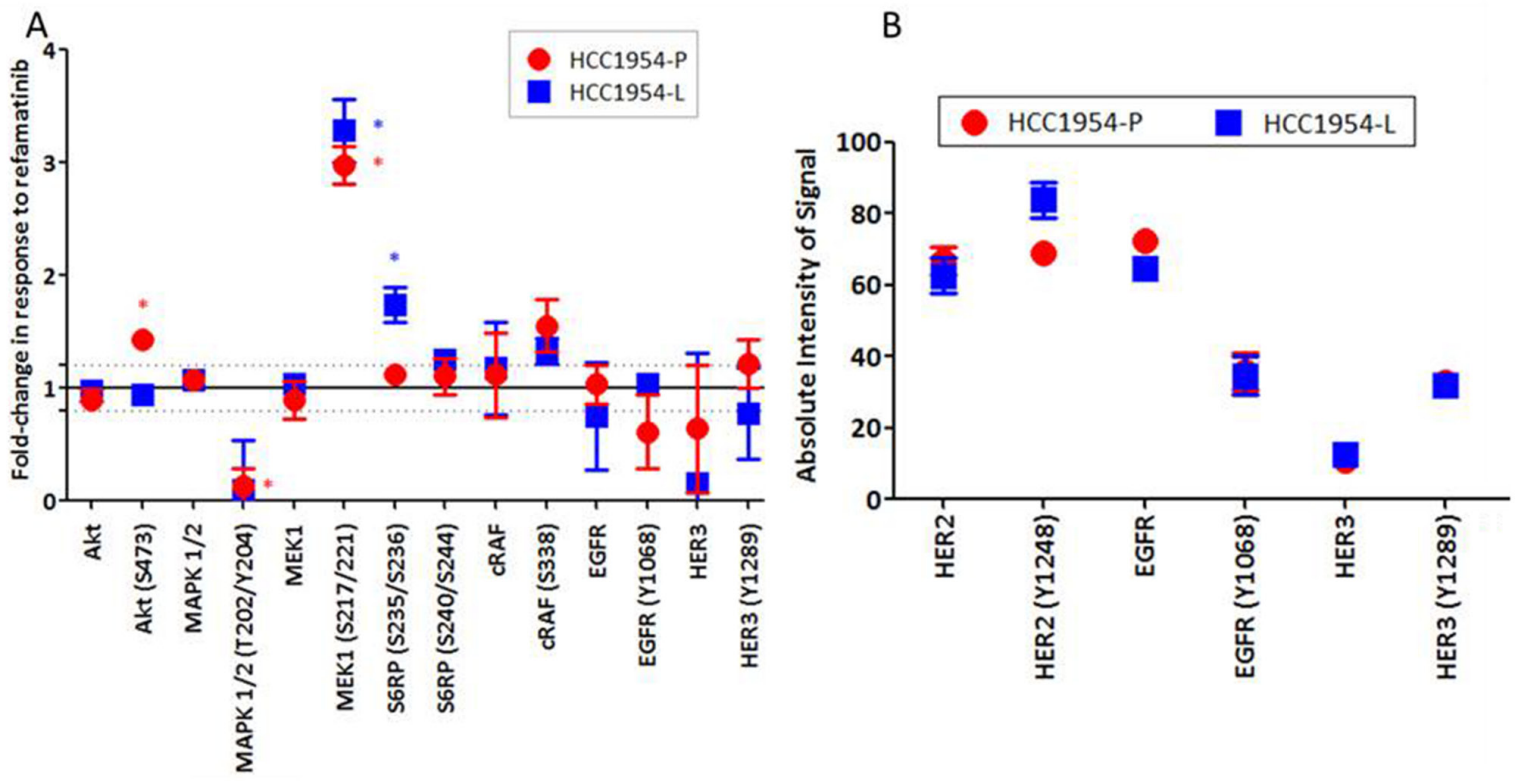
**(A)** RPPA analysis displaying the fold-change in protein expression or phosphorylation relative to untreated controls in cell lines (HCC1954-P, -L) treated with 300nM refametinib (MEKi). **(B)** RPPA analysis displaying the basal levels of EGFR and ERBB3 protein expression and phosphorylation in the HCC1954-P and HCC1954-L cells. Standard deviations are calculated from triplicate independent protein samples analysed on the same RPPA slide. ‘*’ indicates proteins which have a change of signal intensity greater than 1.2 fold and a p-value of <0.05 as determined by the students t-test.

Treatment with refametinib results in a significant increase in MEK1/2 (S217/221) phosphorylation relative to untreated controls in both HCC1954-P (fold change = 2.98 ± 0.29; p=0.012) and -L (acquired lapatinib resistant) cells (fold change = 3.28 ± 0.47; p=0.032) (Figure 2A). However due to the action of refametinib which binds to the ATP binding domain of MEK1/2, the resulting MEK phosphorylation is unable to signal downstream to MAPK. Therefore as expected we see a significant decrease in MAPK ERK1/2 (T202/Y204) phosphorylation in HCC1954-P (fold change = -7.77 ± 2.20; p=0.006) and a close to significant decrease in HCC1954-L (fold change = -9.94 ± 7.47; p=0.069) cells (we also observe a reduction in ERK1/2 (T202Y204) phosphorylation in SKBR3 cells treated with refametinib (Supplementary Figure 2)). In HCC1954-P cells treatment with refametinib results in a potential feedback activation of AKT signalling, as we observed a 1.43 ± 0.11 fold increase in AKT S473 phosphorylation (p=0.018) post treatment, which is not observed in HCC1954-L cells (Figure 2A). Of note, the basal expression and phosphorylation levels of HER2, EGFR and HER3 are similar between HCC1954-P and HCC1954-L cells (Figure 2B). Treatment of HCC1954-L cells with refametinib resulted in a significant 1.74 ± 0.26 fold increase in phosphorylation of S6-Ribosomal Protein (S240/S244) (p=0.04), but this effect was not associated with any increases in expression or phosphorylation of members of the AMPK/mTOR/IGFIR-β signalling pathway (Supplementary Figure 3). Previous studies of ovarian cancer demonstrate a similar effect where increases in MEK phosphorylation are associated with direct phosphorylation of S6-Ribosomal Protein [27], which may result in the inhibition of feedback activation of upstream pathways.

### Proteomic signalling mechanisms underlying cell sensitivity to refametinib

**Figure 3:**
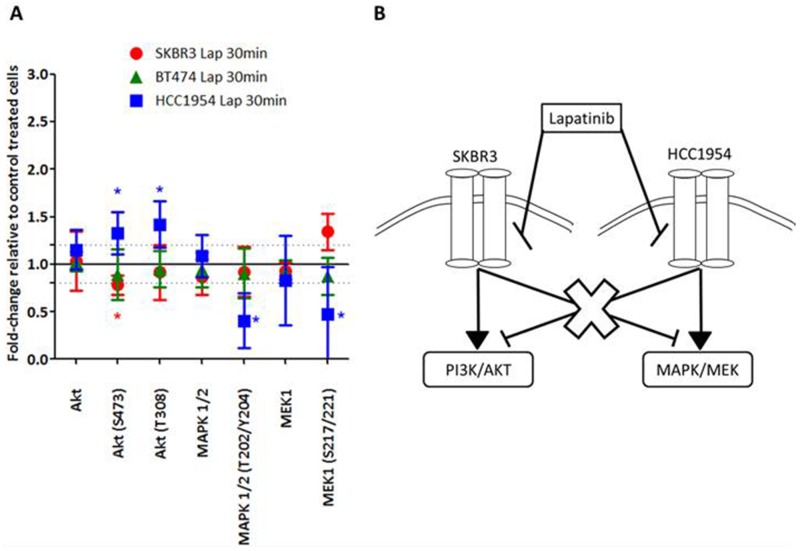
**(A)** RPPA analysis displaying the fold-change in protein expression or phosphorylation relative to control treated cells in cell lines (HCC1954-P, BT474-P and SKBR3-P) treated with 150nM lapatinib for 30 minutes (Lap30min). **(B)** Representative figure demonstrating hypothesised inhibition of MAPK/ERK signalling in HCC1954 and SKBR3 cells as a result of lapatinib treatment. Standard deviations are calculated from triplicate independent protein samples analysed on the same RPPA slide. ‘*’ indicates proteins which have a change of signal intensity of greater than 1.2 fold and a p value of <0.05 as determined by the students t-test.

In refametinib sensitive parental HCC1954-P cells, lapatinib, a dual reversible EGFR/HER2 inhibitor, increased AKT (S473 (fold change = 1.32 ± 0.22; p-value=0.03) and T308 (1.42 ± 0.24; p-value=0.01) phosphorylation whilst decreasing both p-MEK (S217/221) (fold change = -2.10 ± 1.05; p-value=0.02) and p-MAPK (T202/Y204) (fold change = -2.46 ± 0.71; p-value=0.04) phosphorylation (Figure 3A). However in the parental refametinib resistant SKBR3-P cells, treatment with lapatinib had the opposite effect, resulting in a decrease in AKT (S473) phosphorylation (fold change = -1.27 ± 0.13; p-value=0.03), whilst increasing p-MEK (S217/221) phosphorylation (fold change = 1.34 ± 0.19; p-value=0.05). The parental BT474-P cells, with intermediate refametinib sensitivity demonstrate a small non-significant decrease in AKT (S473) and MEK (S217/221) phosphorylation when treated with lapatinib. Thus, in the PIK3CA-mutated refametinib sensitive HCC1954-P cells lapatinib does not decrease AKT phosphorylation, indicating a potential disconnect between HER2 activation and PI3K/AKT signalling, which has been observed previously in breast cancer cell lines [28]. RPPA analysis of the AMPK/mTOR/IGFIR signalling pathway in lapatinib treated HCC1954-P cells indicated that whilst AMPK expression was decreased, there were no associated changes in expression or phosphorylation of either mTOR or IGFIR-β (Supplementary Figure 3). Instead, the decreases in MAPK and MEK phosphorylation in HCC1954-P cells in response to lapatinib suggest that HER2 is ‘connected’ to the MEK/MAPK pathway in this cell line, possibly explaining the refametinib sensitivity. In SKBR3 cells, the opposite occurs in response to lapatinib. The increase in MEK phosphorylation and decrease in AKT phosphorylation caused by lapatinib treatment indicate a potential disconnect between HER2 activation and MEK/MAPK signalling, possibly explaining the resistance of SKBR3 cells to refametinib (Figure 3B).

We thus believe that a HER2-positive breast cancer cells response to lapatinib, where treatment results in a decrease in MEK/MAPK phosphorylation whilst increasing AKT phosphorylation may act as a potential biomarker of sensitivity to refametinib. We believe that this response to lapatinib may indicate that the MEK/MAPK pathway is a major downstream effector of HER2 in these cells. However if AKT and not MEK/MAPK is inhibited by lapatinib, then this may indicate that the AKT pathway and not the MEK/MAPK pathway is a major downstream effector of HER2, thereby identifying a cell that will be resistant to MEKi (in this case SKBR3-P cells).

**Figure 4:**
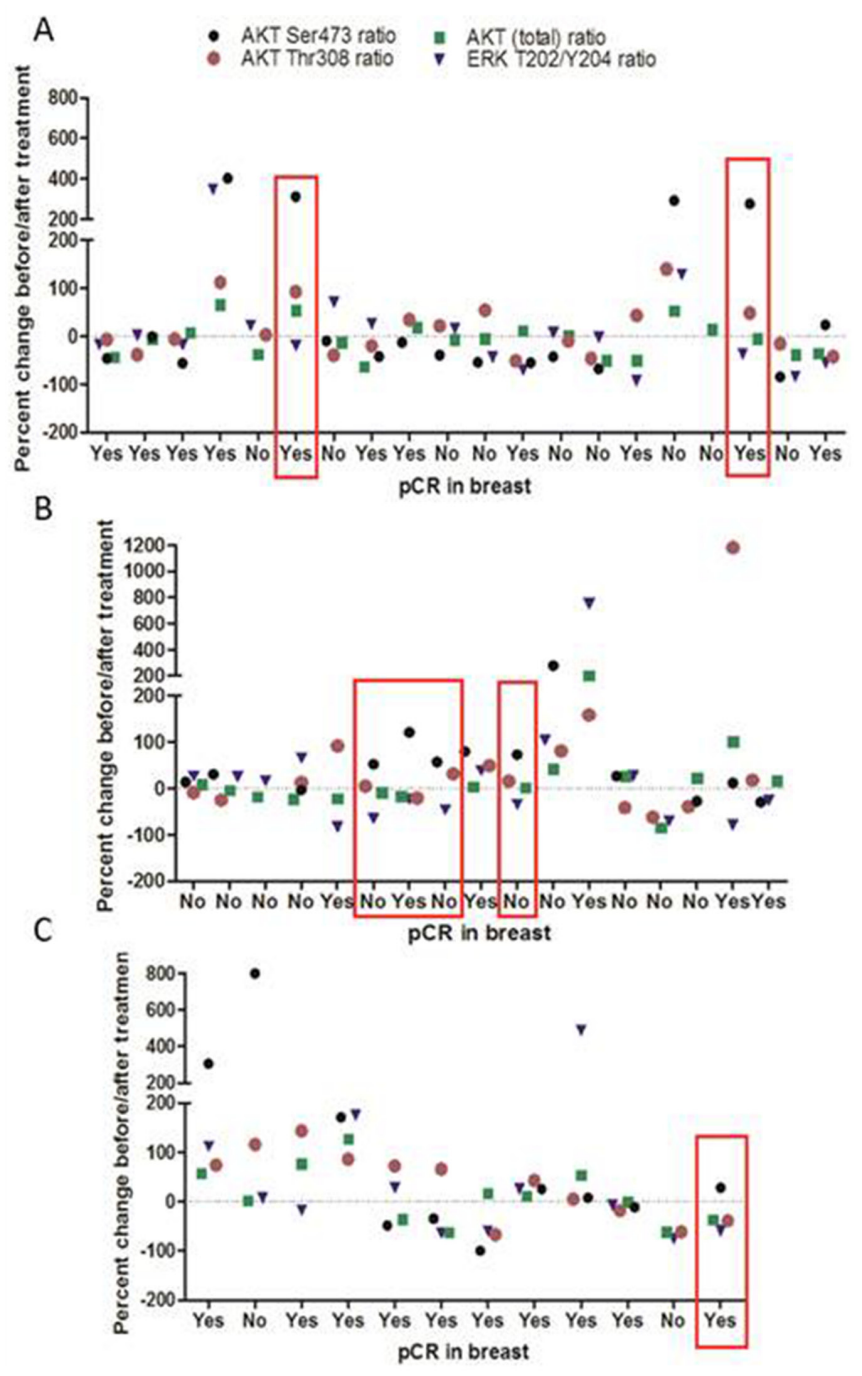
RPPA analysis of the NCT00524303 clinical trial looking at changes in AKT and MAPK signalling pathways in tumours in response to treatment with either **(A)** trastuzumab, **(B)** lapatinib or **(C)** a combination of both. Patient samples where AKT (S473) is increased by >20% and MAPK(T202/Y204) is decreased by >20% are indicated in red.

To determine the frequency *in-vivo* of a HCC1954-type proteomic responses to HER2-targeted therapy, we analysed the RPPA data generated from the NCT00524303 clinical trial, which assessed the impact of treatment with either trastuzumab, lapatinib or a combination of both on signalling alterations in 38 HER2-positive breast cancers (Figure 4). Our analysis identified that 18% (n=7/49) of patient tumours in the NCT00524303 clinical trial, had an increase of AKT (S473) phosphorylation of >20% and a concurrent decrease in MAPK (T202/Y204) phosphorylation of >20% after 14 days of treatment with HER2-targeted therapies. Thus we believe that 18% of HER2-positive breast cancer patients might potentially benefit from refametinib treatment.

### Combinations of refametinib and copanlisib are synergistic in HCC1954 and BT474 parental and lapatinib/trastuzumab resistant HER2-positive breast cancer cells

**Figure 5:**
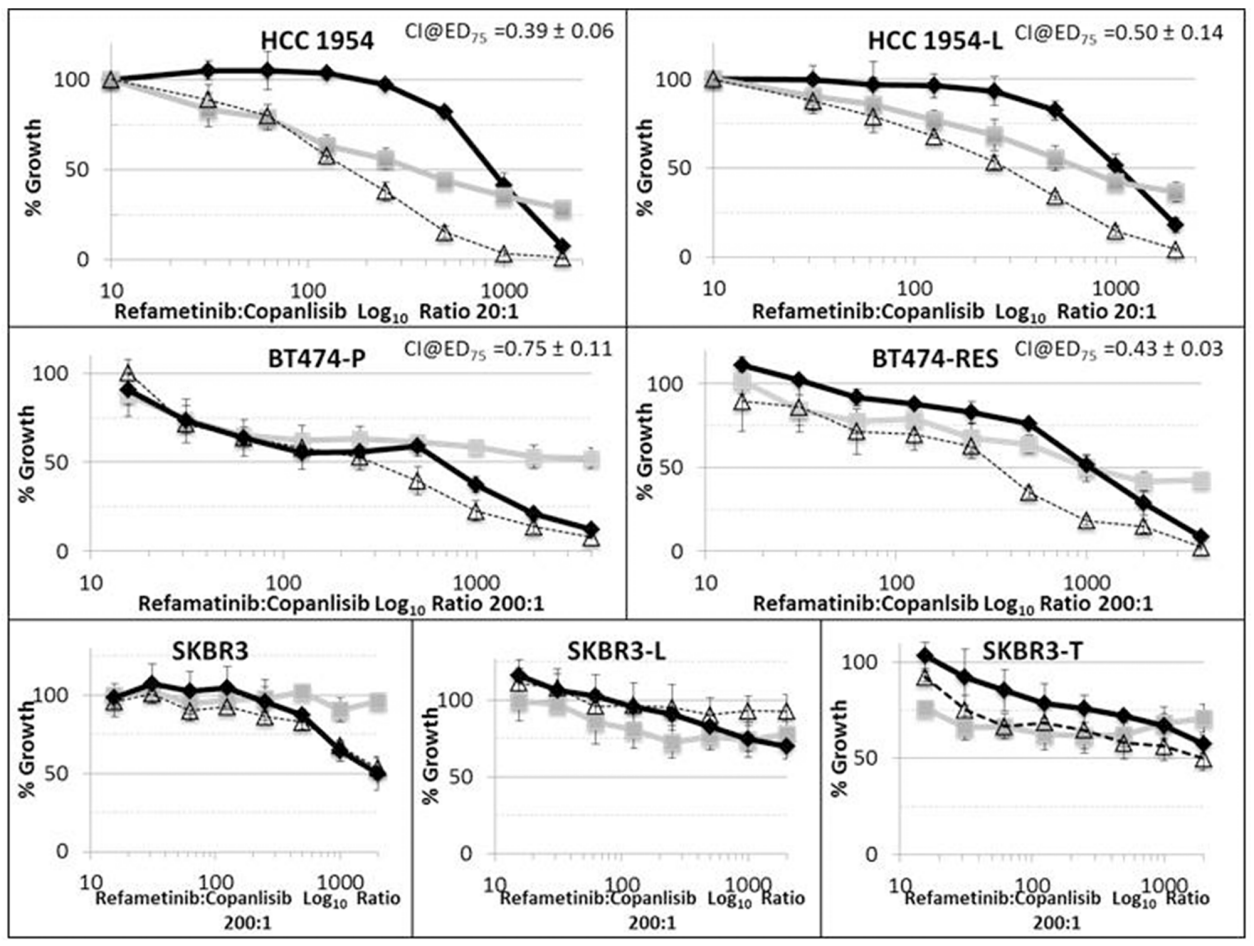
Efficacy of refametinib (MEKi) (-□-), copanlisib (PI3Ki) (-◊-) and a combination of refametinib and copanlisib (--∆--) in a panel of HER2-positive breast cancer cell lines, including parental cells (-P) and those with acquired resistance to either trastuzumab (-T or -Res) or lapatinib (-L) Error bars are representative of standard deviations across triplicate independent experiments. The ratio of refametinib:copanlisib in this assay was fixed at either 20:1 or 200:1.

Combinations of refametinib and the PI3Ki copanlisib enhance growth inhibition relative to testing either drug alone in HCC1954-P, HCC1954-L BT474-P and BT474-RES cell lines (Figure 5). Refametinib in combination with copanlisib induces the strongest anti-proliferative effect in HCC1954-P and BT474-RES cells (Table 2). However there was no enhancement of effect when refametinib and copanlisib were tested in combination in SKBR3 cells. Because refametinib did not achieve an IC_50_ in SKBR3 cells, CI analysis could not be performed in these cell lines [21]. The HCC1954-L cells have a reduced synergism to the combination of drugs relative to the parental HCC1954-P cells, possibly due to significantly elevated p-MAPK (Y202/T204) (p=0.008) and p-MEK (S217/221) (p=0.002) signalling in the HCC1954-L cells (Supplementary Figure 1). The BT474-RES cells have significantly increased p-AKT (S473+T308) levels (both p<0.001) relative to their matched parental cells which may account for the increased sensitivity to the combination of drugs relative to their parental cells and account for the difference in response observed in the HCC1954-L cells.

**Table 2:** Combination Index (CI) values at Effective Dose (ED) _75_ for refametinib (MEKi) combined with copanlisib (PI3Ki) or lapatinib (Lap) in a panel of HER2-positive breast cancer cell lines including parental cells (-P) and matched models of acquired trastuzumab (-RES) or lapatinib (-L) resistance

Cell Line	Refametinib/copanlisib (nM)			Refametinib/ lapatinib (nM)	
	CI @ ED_75_	MEKi IC_50_	PI3Ki:MEKi IC_50_	CI @ ED_75_	Lap:MEKi IC_50_
HCC1954-P	0.39 ± 0.06	357.3 ± 87.8	144.0 ± 27.7	0.39 ± 0.08	127.7 ± 17.8
HCC1954-L	0.50 ± 0.14	713.7 ± 160.2	244.3 ± 37.1	0.58 ± 0.15	317.3 ± 133
BT474-P	0.75 ± 0.11	1245.3 ± 152.0	144.2 ± 16.2	0.80 ± 0.11	19.5 ± 8.6
BT474-RES	0.43 ± 0.03	1379.3 ± 190.5	239.3 ± 101.7	N/A	1374.1 ± 594

Standard deviations are calculated from of triplicate independent experiments. The IC_50_ values shown represent the average value of combining refametinib and copanlisib or combining refametinib and lapatinib together. N/A indicates that a CI value could not be calculated due to both drugs not achieving an IC_50_ in the cell line

**Figure 6:**
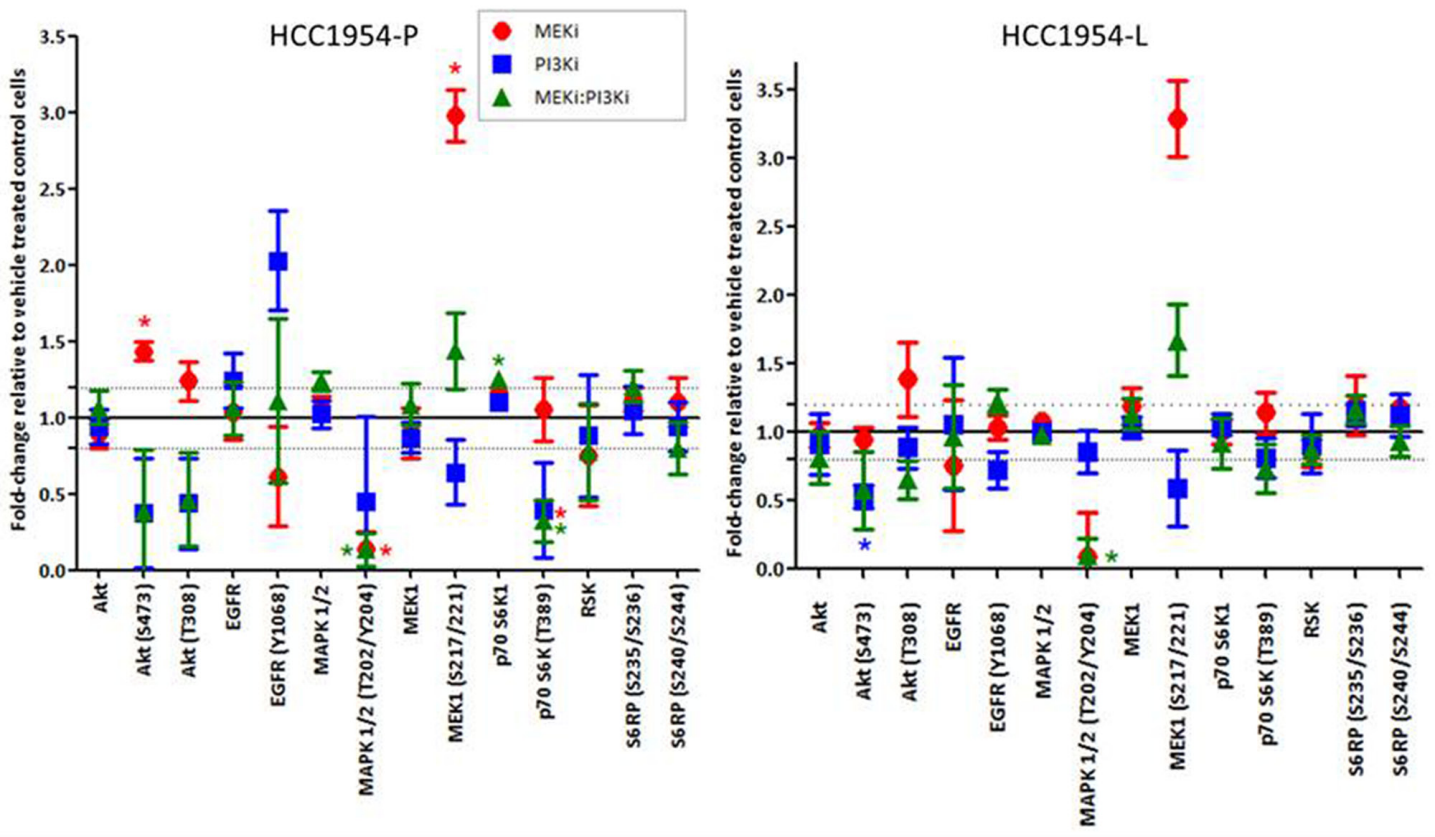
RPPA analysis displaying the fold-change in protein expression or phosphorylation relative to control treated cells in cell lines treated with either 300nM refametinib (MEKi) or 15nM copanlisib (PI3Ki) alone or in combination (MEKi - 300nM: PI3Ki - 15nM) in HCC1954-P (parental) and -L (lapatinib resistant) cells Standard deviations are calculated from at least triplicate biologically independent protein samples analysed on the same RPPA slide. ‘*’ indicates proteins which have a change of signal intensity of greater than 1.2 fold and a p-value of <0.05 as determined by the students t-test.

As shown in Figure 6, and explained in detail previously refametinib treatment of HCC1954-P and -L cells, results in inhibition of MAPK ERK1/2 (T202/Y204) phosphorylation, along with increases in phosphorylation of AKT at S473 and T308 (in HCC1954-P only) and of MEK at S217/221. The later effects are likely mediated via feedback loops. These phosphorylation increases are offset by treatment of these cell lines with copanlisib in addition to refametinib, suggesting a potential mechanism underlying the synergistic augmentation of the anti-proliferative effects of refametinib by copanlisib in these cell lines. In HCC1954-P cells, 30 minutes of treatment with the combination of refametinib and copanlisib resulted in a significant increase in caspase 9 (D330) cleavage (Ref 300nM:Cop 15nM = Fold change 1.20 ± 0.14; p=0.049), indicating that the combination will likely produce a pro-apoptotic effect in these cells (Supplementary Figure 2).

### Combinations of refametinib and lapatinib are synergistic in HCC1954-P, HCC1954-L and BT474-P cells

**Figure 7:**
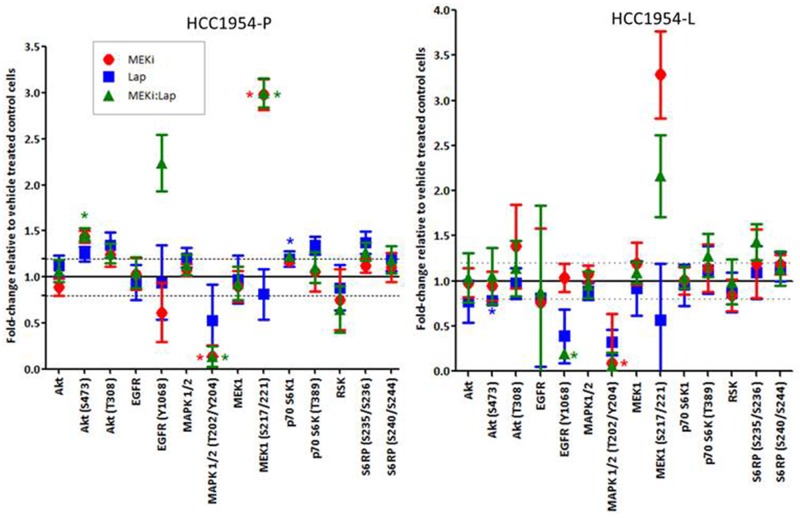
RPPA analysis displaying the fold-change in protein expression or phosphorylation relative to control treated cells in cell lines treated with either 300nM refametinib (MEKi) or Lapatinib (HCC1954-P - 150nM: HCC1954-L - 500nM) alone or in combination in HCC1954-P and -L cells Standard deviations are calculated from at least triplicate biologically independent protein samples analysed on the same RPPA slide. ‘*’ indicates proteins which have a change of signal intensity of greater than 1.2 fold and a p-value of <0.05 as determined by the students t-test.

Combinations of refametinib and lapatinib inhibit growth more effectively than either drug tested alone in HCC1954-P, HCC1954-L and BT474-P cells (Figure 7 and Table 2). Refametinib combined with lapatinib induced the most synergistic anti-proliferative response in HCC1954-P cells. However as shown in Figure 7, refametinib did not augment lapatinib sensitivity in BT474-RES or SKBR3 cells. The reduction in synergism seen in HCC1954-L and BT474-RES cells relative to their matched parental cell lines is again likely due to significantly elevated p-MAPK (Y202/T204) (p=0.008) and p-MEK (S217/221) (p=0.002) signalling in the HCC1954-L cells (Supplementary Figure 1) and significantly increased p-AKT (S473+T308) levels (both p<0.001) in the BT474-RES cells. Because the SKBR3 models did not achieve an IC_50_ to refametinib alone, it was not possible to calculate CI values in these cell lines using the Chou-Talalay equation [21]. In HCC1954-P cells, 30 minutes of treatment with the combination of refametinib and lapatinib resulted in an increase in caspase 9 (D330) cleavage (Ref 300nM:Lap 150nM = Fold change 1.17 ± 0.07; p=0.01), indicating that the combination will likely produce a pro-apoptotic effect in these cells (Supplementary Figure 2), however the result was not significant as the effect did not meet the set parameters (Fold change in expression >1.2 and a p-value of <0.05).

**Figure 8:**
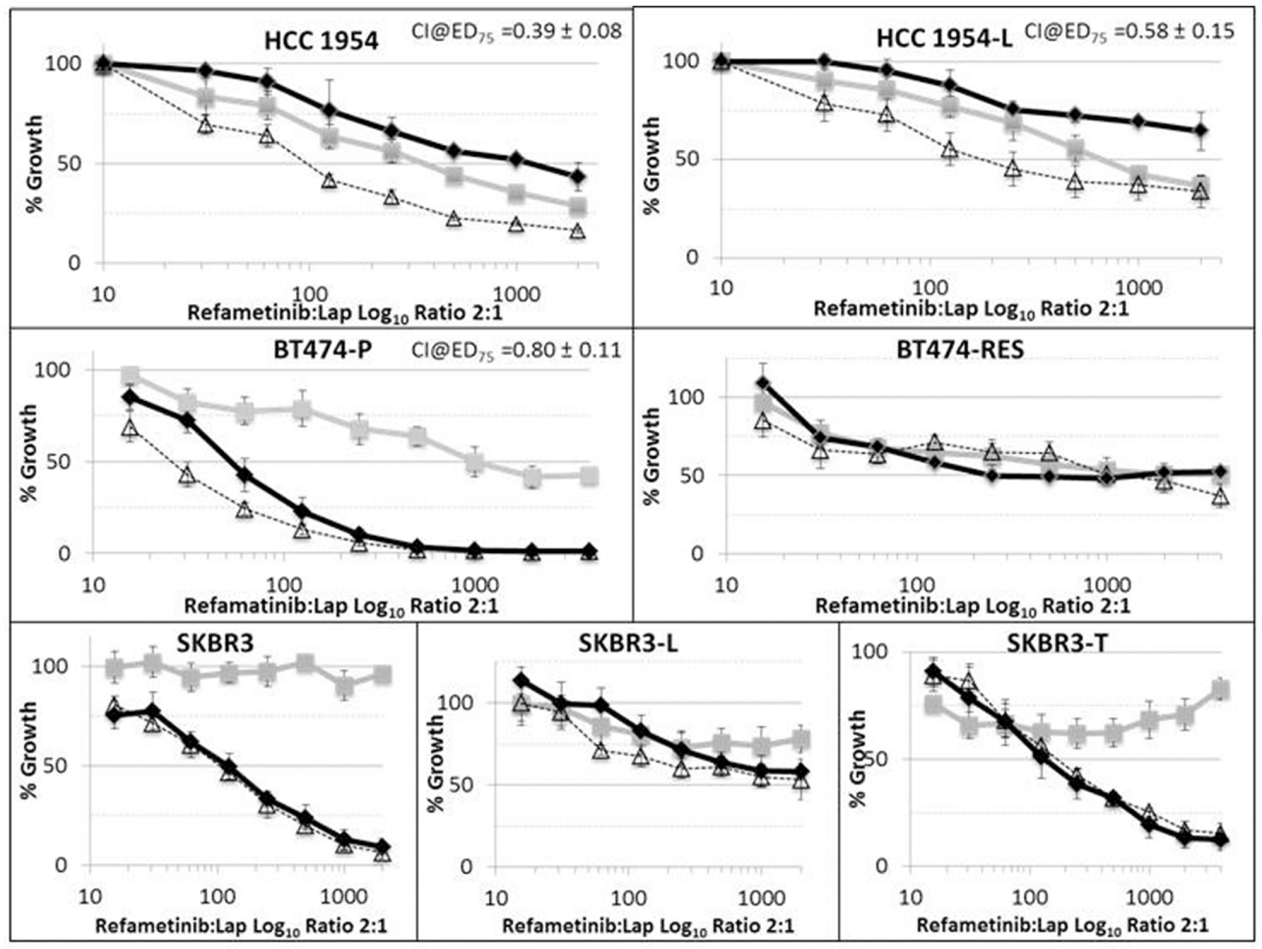
Efficacy of refametinib (MEKi) (-□-), lapatinib (-◊-) and a combination of refametinib and lapatinib (--∆--) in a panel of HER2-positive breast cancer cell lines, including parental cell lines(-P) and matched cells with acquired resistance to either trastuzumab (-T or -Res) or lapatinib (-L) Error bars are representative of standard deviations across triplicate independant experiments. The ratio of refametinib:lapatinib in this assay was fixed at 2:1.

Both lapatinib and refametinib decreased MAPK (but not AKT) phosphorylation in both HCC1954-P and HCC1954-L cells and the combination did not increase this effect (Figure 8) on MAPK (T202/204). As mentioned earlier, this may reflect a preferential modulation of the MAPK but not the AKT signalling pathway by HER2 in HCC1954-P cells. In HCC1954-P cells there was an increase in AKT (S473 and T308) phosphorylation (p=0.055) after treatment with either lapatinib or refametinib alone, again this increase was not increased by combining refametinib and lapatinib. This was not the case in HCC1954-L cells which may have thus adapted their AKT responsiveness to lapatinib and refametinib. Overall, when looking at our selected proteomic biomarkers that reflect PI3K and MAPK signalling, an obvious reason underlying the synergy between lapatinib and refametinib was not apparent in HCC1954-P or -L cells. Refametinib treatment (@300nM for 30 minutes) of HCC1954-P and HCC1954-L cell lines did not significantly alter in an indirect manner the expression or phosphorylation of other proteins from the PIK3/AKT or MEK/MAPK signalling pathways aside from those described above (See full list of antibodies used on RPPA in the Supplementary Table 1).

### Combinations of trastuzumab and refametinib may improve response to either drug tested alone in some HER2-positive breast cancer cells

The combinations of trastuzumab (10μg/ml) and refametinib (250nM (p=0.039) and 500nM (p=0.027)) enhanced response relative to testing either drug alone in BT474-RES cells. However the combination did not result in any enhancement of proliferation inhibition compared to testing either therapy alone in any other cell line tested (Supplementary Figure 4). Because HCC1954-P cells have no innate sensitivity to trastuzumab we did not test the combination of trastuzumab and refametinib in those models, despite them being the most refametinib sensitive cell lines.

## DISCUSSION

Resistance to trastuzumab in the metastatic setting remains a significant clinical problem, with up to 30 % of HER2-positive breast cancer patients not responding [29]. Mutations in PIK3CA occur in approximately 20-30% of HER2-postive breast cancers, and *in vitro* investigation and clinical trials have shown the benefit of targeting the PI3K/AKT pathway in HER2-positive breast cancer as a way of overcoming trastuzumab and lapatinib resistance in some cases [10, 22, 30]. However little attention has been paid to targeting the MEK/MAPK pathway in HER2-positive breast cancer despite the fact that it is also frequently activated in this disease and has been recently established as a potential target for therapy [12]. In fact a recent article by Cheng *et al* (2016) found that PIK3CA mutated HER2 initiated mammary tumours escape PI3K dependency by activating MAPK/ERK signalling [13]. MEK inhibitors including refametinib have been tested in hepatocellular carcinoma, pancreatic, lung and colorectal cancers. In fact current trials of the MEK inhibitor trametinib in triple negative breast cancer are underway (NCT01964924). However to date no-one has studied the role of MEK inhibition in HER2-positive breast cancer despite several cell lines being sensitive to MEK inhibitors (Supplementary Table 3).

MEK inhibitors trametinib and PD-0325901 were found to have antiproliferative effects in triple negative breast cancer cell lines [26, 31–33]. However we observed that HER2-positive breast cancer cell lines also are sensitive to both trametinib and PD-0325901 supporting our belief that MEK inhibitors are interesting targets in HER2-positive breast cancer. RPPA analysis demonstrated that breast cancer cell lines (all subtypes) with higher expression of MEK1, are more likely to be sensitive to the MEK inhibitors trametinib and PD-0325901. MEK is implicated in the regulation of proliferation and breast cancer cells with elevated MEK.

Refametinib (BAY86-9766), a novel potent allosteric MEK1/2 inhibitor from Bayer pharmaceuticals has undergone extensive preclinical testing in solid tumours including breast cancer [25, 34]. It has been shown to inhibit MAPK signalling downstream from MEK *in vitro*. Refametinib has been previously tested both alone [35] and in combination with both the mutli-target tyrosine kinase inhibitor sorafenib [36] and the PI3K inhibitor copanlisib [37] in Phase I and Phase II studies. It was well tolerated in all trials, with manageable drug-related adverse effects, either used alone or in combination with targeted therapies and demonstrated benefit in some patients with advanced cancer [35-38].

Pharmacodynamics analysis of patients recruited to the Phase I trial of refametinib [36] identified that refametinib can achieve a peak plasma conc of 700μg/ml (1.223μM). We classified HCC1954 cells which have an IC_50_ of <500nM as sensitive to refametinib, whilst BT474 cells which have an IC_50_ of 1.2μM are classified as slightly sensitive. SKBR3 cells are insensitive to refametinib at the doses tested. All cell line models in our study were p53 mutated however previous studies by Liu *et al* 2010 [24] in colorectal cancer cell lines demonstrate that refametinib is effective regardless of the p53 status of the cell line models [24]. The BT474-P cell line used in our study was ER positive whilst HCC1954 and SKBR3 cells were ER negative limiting the likelihood of ER being a contributing factor to refametinib sensitivity. Previous data suggest that refametinib is effective independent of PI3K mutational status [23, 24], supported by our findings in HCC1954-P and BT474-P cells which both harbour a PIK3CA mutation.

HCC1954 cells with acquired resistance to lapatinib (HCC1954-L) were somewhat more resistant to refametinib than the HCC1954-P cells possibly due to increased p-MAPK and p-MEK signalling, whilst BT474 cells with acquired resistance to trastuzumab (BT474-RES) had a similar IC_50_ to refametinib relative to the parental cells. Responses to lapatinib, copanlisib and trastuzumab in these cells have already been reported by us [22].

RPPA analysis found that SKBR3-P cells, which are resistant to refametinib, have higher baseline phosphorylation levels of EGFR Y1068 relative to the more sensitive HCC1954-P and BT474-P cell lines. Phosphorylation of EGFR Y1068 leads to GRB2 binding to EGFR which can increase downstream signalling of MEK/MAPK pathway. Treatment of SKBR3 cells with either lapatinib or refametinib did not reduce EGFR Y1068 signalling. In fact Henjes *et al* 2012 [39] identified that cells with elevated EGFR signalling had attenuated response to ERBB2-inhibitors. Therefore the elevated autophosphorylation of EGFR in SKBR3 cells may play a role in refametinib resistance.

RPPA analysis also reveals that single agent refametinib (250nM) completely inhibits MAPK phosphorylation signalling in HCC1954-P and -L cells and SKBR3-P (Supplementary Figure 1) after 30 minutes treatment as expected. However, in HCC1954-P cells refametinib treatment also resulted in a significant increase in p-AKT S473 and T308 signalling identifying a potential link between inhibition of MEK/MAPK signalling and feedback activation of AKT in these cells. No studies of feedback loop activation induced by refametinib have been published; however, previous studies of MEK/MAPK inhibition in BRAF-inhibitor resistant melanoma cells show that MEK inhibitors can activate AKT signalling as an escape mechanism through increased MEK/RAF activation [40]. Another study identified that in B-RAF mutated melanoma cells, MEK inhibitor mediated activation of AKT may be enhanced by increased ERBB3 signalling [41]. In HCC1954, the cells have detectable levels of c-RAF, EGFR and ERBB3 protein expression. However, treatment with refametinib does not significantly change the expression or phosphorylation of any of these proteins, likely identifying that the mechanism for feedback activation of AKT may not be mediated by either c-RAF, EGFR or ERBB3 in this cell line. In HCC1954-L cells treated with refametinib, despite complete inhibition of MAPK phosphorylation, downstream increases in S6 Ribosomal Protein (S240/S244) signalling occur without any associated increases in AKT phosphorylation. Analysis of potential activation of AKT/PI3K signalling in HCC1954-L cells by the mTOR/IGFIR feedback loop identified by Petricoin *et al* 2007 [42] demonstrated that treatment with both lapatinib and rapamycin did not increase phosphorylation of either mTOR or IGFIR However, these data suggest that refametinib sensitivity in HER2-positive breast cancer cells could be limited by feedback loop activation, and that the mechanisms underlying these feedback loops may differ between parental HER2-positive breast cancer cells and cells that have acquired resistance to HER2-inhibitors.

Interestingly we found that the treatment of HCC1954-P cells with lapatinib (dual EGFR/HER2 inhibitor) increased AKT phosphorylation whilst decreasing phosphorylation of both MEK and MAPK. This result was in direct contrast to the results observed in SKBR3 cells after lapatinib treatment. Previous studies demonstrated the dominant HER-dimerization partner for HER2 in HCC1954 cells is EGFR [43]. Network reconstruction analysis of these cells also found that HCC1954 cells likely signal equally through the PI3K and MAPK pathways [43]. Our analysis of lapatinib response in refametinib-sensitive HCC1954-P cells suggests that the MEK/MAPK may be a dominant pathway for HER2-signalling and that HER2 may not directly activate the PI3K/AKT pathway in this cell line. This increase in AKT phosphorylation and decrease in MAPK/MEK phosphorylation in response to lapatinib may act as a sensitivity biomarker for refametinib in HER2-positive breast cancer cell lines, whereby cells which signal primarily through the MAPK pathway are most sensitive to MEKi such as refametinib. In BT474-P cells, with intermediate sensitivity to refametinib, lapatinib treatment does not significantly increase or decrease AKT, MEK or MAPK phosphorylation (Figure 3). Using RPPA analysis of tumours taken from patients included in the NCT00524303 clinical trial, we found that 18% of patient tumours had similar proteomic changes in response to HER2-targeted therapies as observed in the HCC1954-P cells. These patients might thus gain benefit from refametinib therapy although further work is needed to validate this.

We found that combining refametinib with the PI3Ki copanlisib resulted in synergistically greater proliferation inhibition relative to testing either drug alone in both parental HCC1954-P and BT474-P cell lines and in cell lines generated from these to have acquired resistance to lapatinib (HCC1954-L) and trastuzumab (BT474-RES). Combinations of refametinib and copanlisib did not improve the anti-proliferative efficiency of either drug in refametinib resistant SKBR3 cells. RPPA analysis of the proteomic effects of the combination of refametinib and copanlisib in HCC1954-P and HCC1954-L cells revealed complete inhibition of MAPK phosphorylation with the increases in AKT (T308) and MEK phosphorylation induced by refametinib offset by its combination with copanlisib. This effect of the combination on both AKT and MAPK signalling may identify why combinations of PI3Ki and MEKi are equally synergistic in these parental and lapatinib resistant cell models. In HCC1954-P cells the combination of copanlisib and refametinib increased caspase 9 (D330) cleavage, indicating an induction of an apoptotic response in these cells. The hypothesis that combinations of refametinib and copanlisib induces apoptosis in our cells is supported by previous work conducted by Liu *et al* 2010 [23, 24] in both colorectal and lung cancer models who demonstrated that combinations of copanlisib and refametinib increased apoptosis after 48 hours treatment in both cancers.

We also found that combinations of lapatinib and refametinib were synergistic in HCC1954-P and -L as well as parental BT474-P cell lines. However, the combination had little effect in refametinib resistant SKBR3-P, -L and -T cell line models. In HCC1954-P and -L cells the combination of lapatinib and refametinib resulted in significantly better IC_50_ values better than those of lapatinib or refametinib alone. However RPPA analysis using the antibodies in this study was unable to identify a potential reason underlying the synergy between lapatinib and refametinib in HER2-positive breast cancer cell lines. In HCC1954-P cells the combination of lapatinib and refametinib induced an increase in caspase 9 (D330) cleavage, however the effect was not deemed significant as it failed to reach the established parameters.

We have found that refametinib, an extensively studied allosteric MEK1/2 inhibitor, which has undergone extensive preclinical and clinical evaluation in multiple cancer subtypes, has sensitivity in HER2-positive breast cancer cell lines. Greater understanding of the importance of a disconnect between ERBB-family activation and downstream signalling of the PI3K/AKT or MEK/MAPK pathways is required. By strengthening the role of this potential biomarker for refametinib in HER2-postitive breast cancers we will further elucidate the role of MEK inhibitors in the treatment of cancer.

In summary, refametinib has anti-proliferative effects as monotherapy in some HER2-positive breast cancer cells including models of acquired resistance to trastuzumab or lapatinib. Interestingly combinations of refametinib and either lapatinib or copanlisib also induce synergistic anti-proliferative effects in certain HER2-positive breast cancer cell lines. HER2-positive breast cancer cells in which HER2-inhibitors such as lapatinib inhibit MEK/MAP signalling and activate PI3K/AKT signalling may be more likely to be sensitive to the anti-proliferative effects of refametinib. The combination of lapatinib and refametinib also restores the sensitivity to HER2-inhibitors in cells with acquired resistance to lapatinib. These results provide the rationale for testing of MEK inhibitors such as refametinib in patients with HER2-positive breast cancer.

## MATERIALS AND METHODS

### Cell culture

Human HER2-positive breast cancer cell lines (Table 1) were obtained from the National Institute for Cellular Biotechnology (NICB), Dublin City University, and the Division of Haematology/Oncology, University of California, Los Angeles (UCLA). Resistant variants were developed as previously described [14, 15] and BT474-RES (UCLA) was developed by twice weekly dosing of 100 ug/ml trastuzumab for 6 months. All cell lines (Table 1) were grown in RPMI-1640 medium (Sigma) supplemented with 10% FCS and 1% Penicillin/Streptomycin (P/S) and maintained at 37 °C with 5% CO_2_. Cell line identities were confirmed by DNA fingerprinting, which was performed by Source Biosciences (Supplementary Table 2). Cell lines were Mycoplasma tested before and after the *in vitro* experiments. Trastuzumab (21 mg/ml) was obtained from St James University Hospital and prepared in bacteriostatic water. Lapatinib was purchased from Sequoia Chemicals and a stock solution (10.8mM) was prepared in dimethylsulfoxide (DMSO). BAY 80-6946 (copanlisib) (a PI3K inhibitor (PI3Ki)) and BAY86-9766 (refametinib) (a MEK1/2 inhibitor (MEKi)) were obtained under MTA from Bayer pharmaceuticals and stocks (5mM copanlisib; 10mM refametinib) were prepared in 100% DMSO with 10mM TFA, and 100% DMSO respectively. The MEKi GDC-0973 was obtained under MTA from Genentech and stocks (10mM) were prepared in 10% DMSO.

### Proliferation assays

For all resistant cell lines drug was removed from the cells at least 7-days prior to starting assays, and no P/S was added to media during proliferation assays. 3 x 10^3^ cells/well were seeded in 96-well plates, apart from BT474-P and BT474-RES which were seeded at 5 x 10^3^ cells/well. Plates were incubated overnight at 37 °C to allow cells to adhere. Drugs were added to the plates at specific concentrations and incubated at 37 °C. MEKi and lapatinib were combined together in cell lines at a ratio of 2:1, whilst MEKi and PI3Ki were combined at a ratio of either 20:1 in HCC1954 and HCC1954-L cells or 200:1 in SKBR3 or BT474 models. Following 5-day incubation, during which control cells attained 80-90% confluence, all media was removed from the plates, and washed once with PBS. Proliferation was measured using the acid phosphatase assay as previously described [16]. A minimum of triplicate biological assays were carried out for each experiment.

### Protein extraction from cell lines

4 x 10^5^ cells were seeded into 6-well plates, where serum free medium was added to the wells and incubated overnight to synchronise the cells. The following morning cells were treated with the relevant drug and concentration (MEKi - 300nM; PI3Ki - 15nM; lapatinib - HCC1954-P - 150nM; HCC1954-L - 500nM) or a similar concentration of DMSO/DMSO-TFA (vehicle control) in 5% FCS for 30 minutes. To extract protein all media was removed and cells were washed 2X with PBS. 100μl lysis buffer (15% NaCl 1M, 1% triton X-100, 5% Tris, 14% phosphatase inhibitors 7X, 65% dH2O) was added to the plate and cells scraped, with lysates transferred to microcentrifuge tubes and vortexed for ten seconds before being centrifuged at 14,000rpm for 10min at 4°C. Protein was quantified by the biocinchoninic acid (BCA) assay and stored at -80°C.

### Patient samples

LPT109096; NCT00524303 is a phase II study which randomized patients with HER2-positive stage II or III invasive breast cancer to treatment with trastuzumab, lapatinib, or both together with chemotherapy. All data were verified by US Oncology Research and GlaxoSmithKline (US Oncology 05-074, GlaxoSmithKline LPT109096, registration NCT00524303). This study was developed by the Breast Committee of US Oncology Research with GlaxoSmithKline and, in accordance with the precepts of the Helsinki Declaration, was approved by the US Oncology Research central institutional review board, Houston, TX, and clinically performed by US Oncology Research.

Core breast biopsies in the surgeon’s office were collected at baseline and after the first 14 days of anti-HER2 treatment, prior to initiation of chemotherapy: 4 cores at each time point. One core from each time point was fixed in a protein/phosphoprotein preservative developed by George Mason University, Manassas, VA [14]. Frozen tissue sections were prepared for laser capture microdissection of breast tumour and/or stroma with analysis by reverse phase protein microarray. The protein endpoints evaluated are listed in Supplementary Table 1.

### Reverse phase protein array analysis (RPPA)

RPPA on the clinical samples was performed as previously described [17]. RPPA analysis of the *in vitro* data was performed as previously described by us [18, 19]. The complete RPPA methods and a full list of antibodies used are in the Supplementary Table 1. RPPA analysis was carried out using triplicate biological replicates, and the data was normalised by protein loading using the entire antibody panel. RPPA sensitivity is a function of antibody affinity and protein concentration. Based on our internal precision studies [20], we can detect changes in protein expression with a CV of less than 20%. We also ensured that all results must have a p-value as calculated by the students t-test of <0.05 to be determined as significant.

### Western blotting

Loading buffer was made up and added to each protein sample. 10 cm Novex® and NuPAGE® Mini Gels with the Bolt® Mini Gel Tank were run at 130V for 35 minutes as outlined by the supplied procedure. The protein was transferred to Hybond-ECL nitrocellulose membrane (Amersham Biosciences) using a semi-dry transfer unit (Atto). The membrane was blocked with 5% milk powder (Biorad) in 0.1% TBS-Tween at room temperature for 1 hour, then exposed to primary antibody with 0.1% TBS-Tween in 5% milk powder for 2 hours. The membrane was washed three times with 0.5% TBS-Tween and then incubated at room temperature with secondary antibody in 5% milk powder with 0.5% TBS-Tween for 1 hour. The membrane was washed three times with 0.5% TBS-Tween followed by one wash with TBS alone. Detection was performed using Luminol (Santa Cruz Biotechnology).

### Statistical analysis

IC_50_ and combination index (CI) values @ Effective Dose _75_ (ED_75_) were calculated using CalcuSyn software (BioSoft). A CI value of < 0.9 is considered synergistic, 0.9 - 1.1 is considered additive and > 1.1 is considered antagonistic [21]. The Student’s *t-*test was used to evaluate and compare the effects of refametinib, copanlisib and lapatinib alone and in combination on protein expression and phosphorylation in our RPPA data. A Kruskal-Wallis non-parametric test was performed to compare the anti-proliferative effects of trastuzumab alone, BAY 80-6946 alone and the combination. *P* < 0.05 was considered statistically significant.

## SUPPLEMENTARY MATERIALS FIGURES AND TABLES




