# Supplementary material for: A preclinical evaluation of the MEK inhibitor refametinib in HER2-positive breast cancer cell lines including those with acquired resistance to trastuzumab or lapatinib
Source: Oncotarget. 2017 Jul 22;8(49):85120–35. doi: 10.18632/oncotarget.19461 (PMC5689598; doi:10.18632/oncotarget.19461)
Supplement: Supplementary file 1 [file oncotarget-08-85120-s001.pdf]

A preclinical evaluation of the MEK inhibitor refametinib in HER2-positive breast cancer cell lines including those with acquired resistance to trastuzumab or lapatinib

SUPPLEMENTARY MATERIALS

Reverse phase protein array (RPPA)

RPPA was carried out as previously described by us [10, 18]. Protein extraction lysates were normalized to 1.5 µg/µL concentration as assessed by bicinchoninic acid assay (DC Protein Assay - Bio-Rad, CA, USA). 3 parts of cell lysates were mixed with 1 part of an SDS buffer (40% Glycerol, 8% SDS, 0.25M Tris-HCL, pH 6.8 plus 2-mercaptoehtanol at 1/10 of the volume) and boiled. Lysates were manually diluted in four-fold serial dilutions with lysis buffer.

A 2470 Arrayer (Aushon BioSystem, MA, USA), created a sample array on Oncyte Avid nitrocellulose-coated slides (Grace Bio-Labs, OR, USA). The slides were stored with desiccant (Drierite, OH, USA) at −20 °C prior to immunostaining.

Immunostaining was performed on an automated slide stainer (Dako Link 48 - Dako, CA, USA) according to the manufacturer's instructions (CSA kit - Dako, CA, USA). Each slide was incubated with a single primary antibody (see Table 1 for details) at room temperature for 30 min. Secondary antibody was goat anti-rabbit IgG (1:5000) (Vector Laboratories, CA, USA) or rabbit anti-mouse IgG (1:10) (Dako, CA, USA). Dako Secondary antibodies were used as a starting point for amplification via horseradish peroxidase-mediated biotinyl tyramide with chromogenic detection (diaminobenzidine) according to the manufacturer's instructions (Dako, CA, USA).

Scanned TIFF images of slides were analyzed using Microvigene software version 5.1 (VigeneTech Inc., MA, USA) to generate spot signal intensities [44]. Instead of generating multiple linear regression curves for data quantification over each series of serial dilutions, the QRPPA module of Microvigene using a 4 parameter logistic-log model ("SuperCurve" algorithm [45]) that uses all spots within one array to form a sigmoid antigen-binding kinetic curve.

Finally the spots are normalized by protein loading using the entire panel of antibodies. Briefly, normalization is processed as follows: we determined the median for each antibody across the sample set and we divided each raw linear value by the median within each antibody to get the median-centered ratio. After that, we calculated the median from median-centered ratio for each sample across the entire panel of antibodies. This median functions as a correction factor (CF) for protein loading adjustment. We considered the samples an outlier if the CF is above 2.5 or below 0.25. Finally, we divided the raw data in linear value by the CF to obtain the normalized value.

Calculation for fold change in protein expression phosphorylation

The formula to calculate fold change is calculated according to the error of propagation,

$$Q = \frac{ab\cdots c}{xy\cdots z},$$

The formula to calculate the standard deviation according to the error of propagation is,

$$\frac{\delta v}{v} = \sqrt{\left(\frac{\delta d}{d}\right)^2 + \left(\frac{\delta t}{t}\right)^2}$$

http://ipl.physics.harvard.edu/wp-uploads/2013/03/PS3_Error_Propagation_sp13.pdf

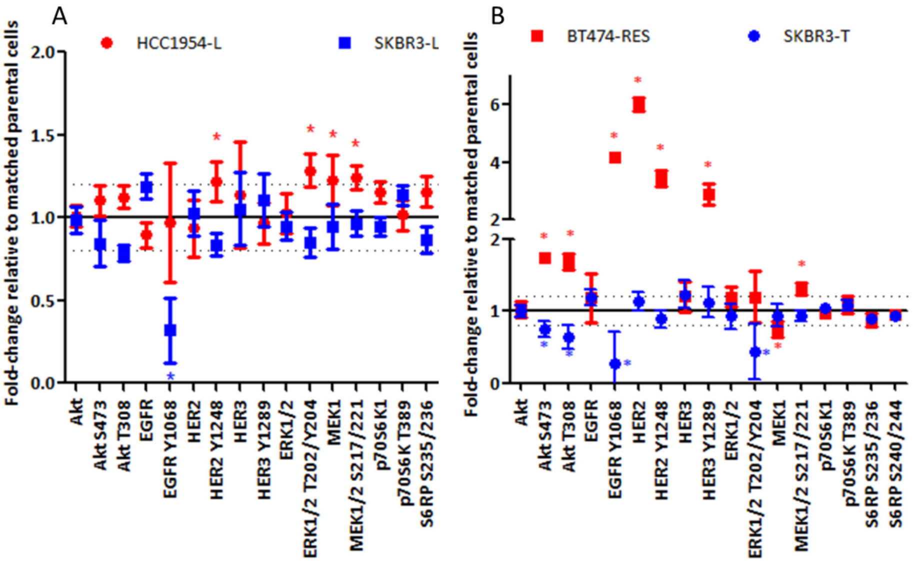

Supplementary Figure 1: Fold change in expression and phosphorylation of proteins in the lapatinib resistant (HCC1954-L, SKBR3-L) and trastuzumab resistant (SKBR3-T, BT474-RES) cells relative to their matched parental cell lines. Standard deviations are calculated from triplicate independent protein samples analysed on the same RPPA slide. '*' indicates proteins which have a change of signal intensity of greater than 20% and a p-value of <0.05 as determined by the students t-test.

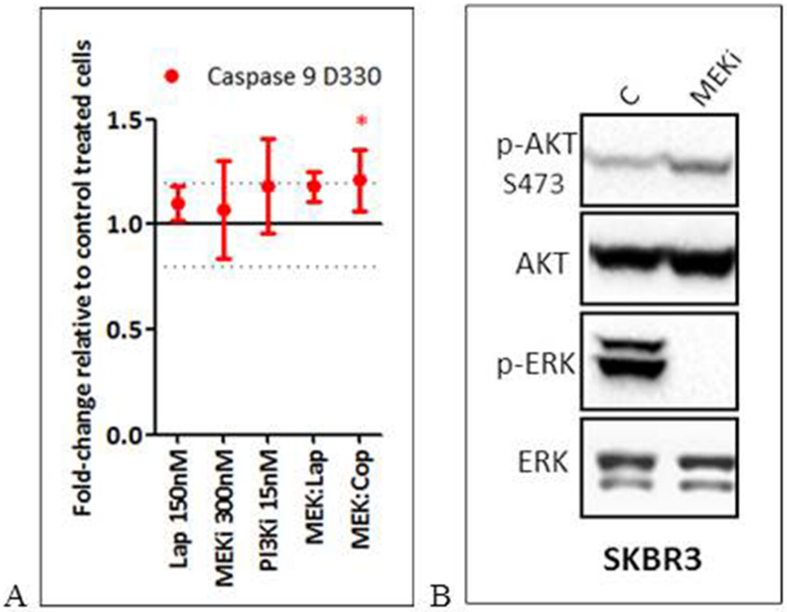

Supplementary Figure 2: (A) Fold change in caspase 9 D330 cleavage after treatment of HCC1954-P cells with lapatinib, refametinib and copanlisib used alone or in combination. Standard deviations are calculated from triplicate independent protein samples analysed on the same RPPA slide. '*' indicates proteins which have a change of signal intensity of greater than 20% and a p-value of <0.05 as determined by the students t-test. **(B)** Western analysis of AKT, p-AKT (S473), ERK, p-ERK (T202/204), expression in refametinib treated SKBR3-P cells.

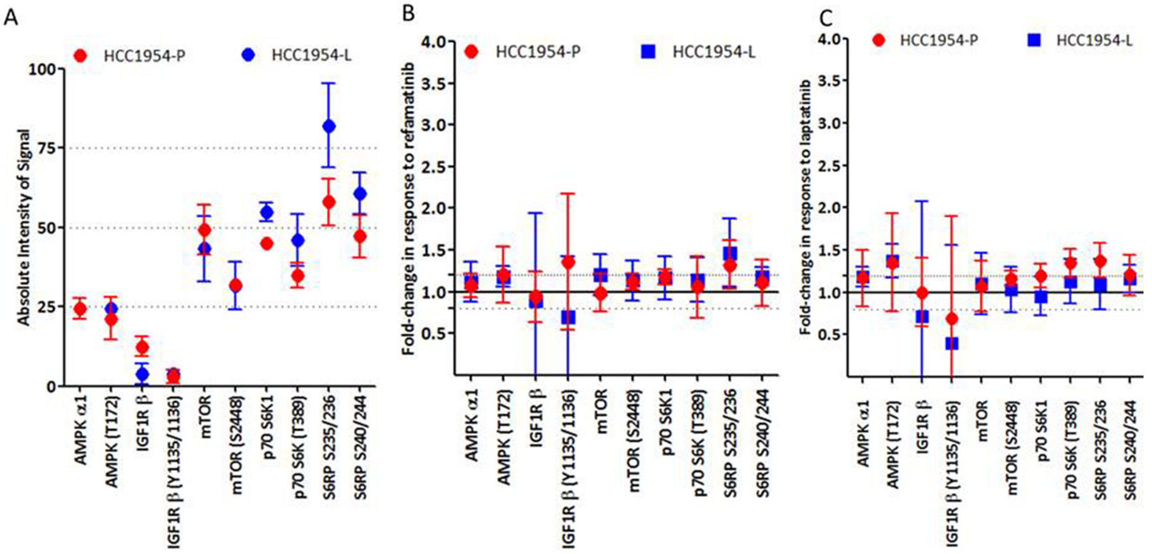

Supplementary Figure 3: Analysis of refametinib induced activation of AKT through AMPK/mTOR feedback. (A) Baseline expression of members of AMPK/mTOR signalling pathway in HCC1954-P and HCC1954-L cells. **(B)** Fold-change in expression and phosphorylation of members of the AMPK/mTOR signalling pathway in response to refametinib in HCC1954-P and -L cells **(C)** Fold-change in expression and phosphorylation of members of the AMPK/mTOR signalling pathway in response in lapatinib in HCC1954-P and -L cells. Error bars are representative of standard deviations from triplicate independent experiments.

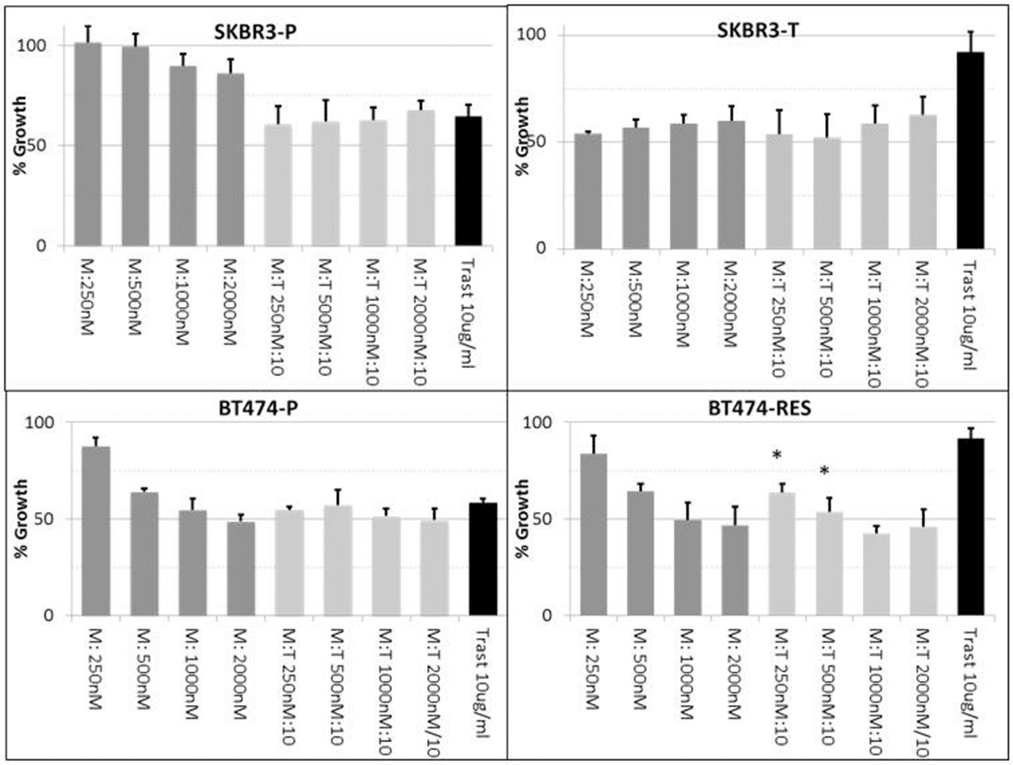

Supplementary Figure 4: The efficacy of combining trastuzumab (T) at 10ng/ml and refametinib (M) at varying concentrations in a panel of HER2-positive breast cancer cell lines including matched models of acquired trastuzumab resistance. Standard Deviations are representative of independent triplicate experiments. '*' indicates a p-value < 0.05 as calculated by Kruskal-Wallis non-parametric test.

Supplementary Table 1: The full list of antibody endpoints used in the RPPA experiments, including the company from which it was purchased including the relevant catalogue number, host species and dilution at which it was used

	Antibody	Cat#	Company	Dil	Host
1	Akt	4691	CellSig	1:3000	R
2	AKT (S473)	9271	CellSig	1:250	R
3	AKT (T308)	2965	CellSig	1:500	R
4	AMPK (T172)	2535	CellSig	1:250	R
5	AMPKalpha1	2532	CellSig	1:250	R
6	C-Raf	04-739	Millipore	1:250	R
7	c-Raf (S338)	9427	CellSig	1:200	R
8	EGFR	2232	CellSig	1:100	R
9	EGFR (Y1173)	1124	Epitomics	1:50	R
10	EGFR (Y992)	2235	CellSig	1:100	R
11	EGFR (Y1068)	2234	CellSig	1:100	R
12	HER2	MS-325-P1	Lab Vision	1:1000	M
13	HER2 (Y1248)	06-229	Upstate	1:750	R
14	HER3	285	Santa Cruz	1:500	R
15	HER3 (Y1289)	4791	CellSig	1:50	R
16	IGFIR-Beta	3027	CellSig	1:500	R
17	IGFIR-Beta (Y1135/1136)	3024	CellSig	1:500	R
18	MAPK - ERK 1/2	9102	CellSig	1:200	R
19	MAPK (T202/Y204) -ERK1/2	4377	CellSig	1:1200	R
20	MEK1	1235-1	Epitomics	1:1200	R
21	MEK1/2 (S217/221)	9154	CellSig	1:1000	R
22	mTOR	2983	CellSig	1:400	R
23	mTOR (S2448)	2971	CellSig	1:100	R
24	p38 MAP Kinase (T180/Y182)	9211	CellSig	1:250	R
25	p38_MAPK	9212	CellSig	1:300	R
26	p70 S6 Kinase	1494-1	Epitomics	1:250	R
27	p70 S6 Kinase (T389)	9205	CellSig	1:250	R
28	S6 Ribosomal Protein (S235/236) (2F9)	4856	CellSig	1:200	R
29	S6 Ribosomal Protein (S240/244)	2215	CellSig	1:3000	R

Supplementary Table 2: DNA fingerprint analysis of cell lines used in this study conducted by Source Bioscience

Sample	Allele	AMEL	CSF1PO	D13S317	D16S539	D18S51	D21S11	D3S1358	D5S818	D7S820	D8S1179	FGA	Penta_D	Penta_E	TH01	TPOX	vWA
**BT474**	**Allele 1**	X	10	11	9	13	28	17	11	9	10	22	9	5	7	8	15
	**Allele 2**	X	11	11	11	18	32.2	17	13	12	12	25	14	5	7	8	16
**ATCC reference**	**Allele**	X	10,11	11	9,11	N/A	N/A	N/A	11,13	9,12	N/A	N/A	N/A	N/A	7	8	15,16
**BT474-RES**	**Allele 1**	X	10	11	9	13	28	17	11	9	10	22	9	5	7	8	15
	**Allele 2**	X	11	11	11	18	32.2	17	13	12	12	25	14	5	7	8	16
**ATCC reference**	**Allele**	X	10,11	11	9,11	N/A	N/A	N/A	11,13	9,12	N/A	N/A	N/A	N/A	7	8	15,16
**HCC1954-PAR**	**Allele 1**	X	10	8	9	14	28	15	11	10	12	22	9	12	6	8	18
	**Allele 2**	X	10	9	11	18	32.2	16	11	11	15	23	12	16	7	9	19
**ATCC reference**	**Allele**	X	10	8,9	9,11	N/A	N/A	N/A	11	10,11	N/A	N/A	N/A	N/A	6,7	8,9	18,19
**HCC1954-LAP**	**Allele 1**	X	10	8	9	18	28	15	11	10	12	22	9	12	7	8	18
	**Allele 2**	X	10	9	11	18	32.2	16	11	11	15	23	12	16	7	9	19
**ATCC reference**	**Allele**	X	10	8,9	9,11	N/A	N/A	N/A	11	10,11	N/A	N/A	N/A	N/A	6,7	8,9	18,19
**SKBR3-PAR**	**Allele 1**	X	12	11	9	10	30	17	9	9	11	20	9	11	6	10	17
	**Allele 2**	X	12	12	9	13	30.2	17	12	12	12	20	12	11	6	10	18
**ATCC Reference**	**Allele**	X	12	11,12	9	N/A	N/A	N/A	9,12	9,12	N/A	N/A	N/A	N/A	6	10	17,18
**SKBR3-T**	**Allele 1**	X	12	11	9	10	30	17	9	9	11	20	9	10	8	8	17
	**Allele 2**	X	12	12	9	13	30.2	17	12	12	12	20	12	11	9	11	17
**ATCC Reference**	**Allele**	X	12	11,12	9	N/A	N/A	N/A	9,12	9,12	N/A	N/A	N/A	N/A	8,9	8,11	17,18
**SKBR3-L**	**Allele 1**	X	12	11	9	10	30	17	9	9	11	20	9	10	8	8	17
	**Allele 2**	X	12	12	9	13	30.2	17	12	12	12	20	12	11	9	11	17
**ATCC Reference**	**Allele**	X	12	11,12	9	N/A	N/A	N/A	9,12	9,12	N/A	N/A	N/A	N/A	8,9	8,11	17,18

Supplementary Table 3: Comparison of IC_{50} values for MEK inhibitors PD0325901, refametinib (RDEA119), selumitinib (AZD6244), and tremetinib (GSK1120212) in a panel of breast cancer cell lines which are divided relative to their subtype and ERBB2-amplification and ER status. IC_{50} values are taken from GDSC database [1], Garnett et al [2], CCLE database [3] and Daemen et al [4].

See Supplementary File 1
