# Supplementary material for: A preclinical evaluation of the MEK inhibitor refametinib in HER2-positive breast cancer cell lines including those with acquired resistance to trastuzumab or lapatinib
Source: Oncotarget. 2017 Jul 22;8(49):85120–35. doi: 10.18632/oncotarget.19461 (PMC5689598; doi:10.18632/oncotarget.19461)
Supplement: Supplementary file 2 [file oncotarget-08-85120-s002.docx]

Supplementary Table 3: Comparison of IC_50_ values for MEK inhibitors PD0325901, refametinib (RDEA119), selumitinib (AZD6244), and tremetinib (GSK1120212) in a panel of breast cancer cell lines which are divided relative to their subtype and ERBB2-amplification and ER status. IC_50_ values are taken from GDSC database [1], Garnett et al [2], CCLE database [3] and Daemen et al [4].

|  |  |  |  | **PD-0325901 (μM)** | | | **Refametinib (μM)** | | **Selumitinib (μM)** | | | | **Tremetinib (μM)** | |
| --- | --- | --- | --- | --- | --- | --- | --- | --- | --- | --- | --- | --- | --- | --- |
| **Cell line** | **Subtype** | **ERBB2 status** | **ER Status** | GDSC ^[1]^ | Garnett ^[2]^ | CCLE ^[3]^ | Garnett ^[2]^ | GDSC ^[1]^ | GDSC ^[1]^ | Garnett ^[2]^ | CCLE ^[3]^ | Daemen^[4]^ | GDSC ^[1]^ | Daemen^[4]^ |
| BT20 | Basal | Basal | Negative | 0.62 | 1.41 | 8 | 21.45 | 9.46 | 17.70 | 23.26 | 8 | 50 | 0.47 |  |
| DU-4475 | Basal | Basal | Negative | 0.003 | 0.004 |  | 0.036 | 0.047 | 11.600 | 0.044 |  |  | 0.001 |  |
| HCC1143 | Basal | Basal | Negative | 0.21 |  |  |  | 1.25 | 69.50 |  |  | 50 | 0.07 | 16.70 |
| HCC1187 | Basal | Basal | Negative | 0.80 | 5.42 | 8 | 38.08 | 11.70 | 13.60 | 26.86 | 8 | 50 | 6.02 | 16.70 |
| HCC1806 | Basal | Basal | Negative |  | 0.84 | 8 | 41.75 |  |  | 33.27 | 8 | 50 |  | 0.41 |
| HCC1937 | Basal | Basal | Negative | 0.27 | 0.54 |  | 216.47 | 10.10 | 62.80 | 191.99 |  | 50 | 1.03 | 16.70 |
| HCC2157 | Basal | Basal | Negative | 2.24 |  |  |  | 18.30 | 31.20 |  |  |  | 14.60 |  |
| HCC70 | Basal | Basal | Negative |  | 0.15 | 1.25 | 23.33 |  |  | 3.49 | 8 | 1.79 | 0.59 | 0.01 |
| HDQ-P1 | basal | basal | Negative | 0.27 |  | 5.74 |  | 2.13 | 3.96 |  | 8 |  | 1.48 |  |
| Hs 739.T | Basal | Basal | Negative |  |  | 8 |  | 1.38 |  |  | 8 |  |  |  |
| MDAMB468 | Basal | Basal | Negative | 0.07 |  | 8 |  | 5.14 | 14.90 |  | 8 |  | 1.11 | 14.50 |
| COLO824 | Basal | Basal | Negative | 4.06 | 23.52 |  | 11.21 | 3.50 | 40.50 | 255.30 |  |  | 3.18 |  |
| CAL120 | Basal | Basal | Negative | 2.12 | 6.98 |  | 6.44 | 5.34 | 9.36 | 20.06 |  |  | 0.22 |  |
| CAL51 | Basal | Basal | Negative | 1.12 | 13.72 |  | 7.99 | 3.65 | 20.60 | 127.04 |  |  | 1.73 |  |
| CAL851 | Basal | Basal | Negative | 0.14 | 0.24 |  | 3.67 | 2.64 | 2.80 | 4.86 | 3.01 |  | 0.17 |  |
| BT549 | Claudin-low | Claudin-low | Negative | 3.11 | 6.18 | 8 | 19.27 | 13.30 | 65.40 | 85.76 | 8 | 50 | 0.68 | 2.84 |
| HCC1395 | Claudin-low | Claudin-low | Negative | 0.56 | 14.65 | 8 | 62.53 | 17.80 | 46.90 | 117.63 | 8 | 37.80 | 0.89 | 0.19 |
| HCC38 | Claudin-low | Claudin-low | Negative | 3.22 | 21.02 |  | 165.42 | 33.50 | 64.10 | 477.88 |  | 50 | 3.15 | 16.70 |
| HS578T | Claudin-low | Claudin-low | Negative | 0.02 |  | 8 |  |  | 65.00 |  | 8 | 17.80 | 0.55 | 0.03 |
| MDAMB157 | Claudin-low | Claudin-low | Negative | 2.88 | 12.35 | 8 | 21.64 | 7.58 | 24.00 | 126.66 | 8 | 50 | 14.60 | 0.27 |
| MDAMB231 | Claudin-low | Claudin-low | Negative | 0.13 | 0.16 |  | 1.12 | 1.17 | 3.14 | 5.53 |  | 50 | 0.06 | 0.14 |
| MDAMB436 | Claudin-low | Claudin-low | Negative | 0.08 |  | 8 |  | 3.35 | 109.00 |  | 8 |  | 0.51 | 15.50 |
| EVSAT | Luminal | ERBB2-amp | Negative | 0.59 |  |  |  | 25.30 | 32.00 |  |  |  | 4.34 |  |
| HCC2218 | Luminal | ERBB2-amp | Positive | 6.85 |  |  |  | 55.70 | 81.00 |  |  |  | 23.30 |  |
| HCC1569 | Basal | ERBB2-amp | Negative | 0.66 | 2.48 | 8 | 18.89 | 16.10 | 55.20 | 152.40 | 8 | 50 | 0.51 | 16.70 |
| HCC1954 | Basal | ERBB2-amp | Negative | 0.34 | 0.55 | 8 | 30.28 | 13.90 | 7.33 | 13.01 | 8 | 1.46 | 0.17 | 0.16 |
| JIMT1 | Basal | ERBB2-amp | Negative | 0.26 |  |  |  | 5.38 | 26.00 |  |  |  | 0.20 |  |
| AU565 | Luminal | ERBB2-amp | Negative | 0.17 | 5.69 | 8 | 393.62 | 40.10 | 68.10 | 384.67 | 8 | 50 |  | 9.71 |
| MDA-MB-330 | luminal | ERBB2-amp | Negative | 2.30 |  |  |  | 110.00 | 20.10 |  |  |  | 7.60 |  |
| SKBR3 | Luminal | ERBB2-amp | Negative |  |  | 8 | 435.45 | 38.80 |  |  | 8 | 50 |  | 16.70 |
| OCUB-M | Luminal | ERBB2-amp | Negative |  | 0.76 |  |  |  | 6.40 | 12.31 |  |  | 4.82 |  |
| BT474 | Luminal | ERBB2-amp | Positive | 2.01 | 6.67 | 8 | 39.35 | 13.20 | 68.40 | 189.13 | 8 | 50 | 17.60 | 16.70 |
| EFM192A | Luminal | ERBB2-amp | Positive | 0.78 |  |  |  | 27.10 | 13.50 |  |  |  | 4.71 |  |
| HCC1419 | Luminal | ERBB2-amp | Positive | 0.14 |  |  |  | 105.00 | 16.60 |  |  | 17.90 | 11.90 | 0.06 |
| HCC202 | Luminal | ERBB2-amp | Positive | 6.64 |  |  |  | 152.00 | 127.00 |  |  |  | 14.70 | 0.52 |
| MDAMB361 | Luminal | ERBB2-amp | Positive | 0.38 | 0.89 |  | 59.83 | 5.53 | 22.30 | 90.16 |  | 50 | 4.38 | 16.70 |
| UACC812 | Luminal | ERBB2-amp | Positive |  |  | 0.04 |  |  |  |  | 8 | 50 |  | 16.70 |
| UACC893 | Luminal | ERBB2-amp | Positive | 4.40 | 8.99 |  | 24.28 | 15.30 |  |  |  |  | 0.29 | 1.68 |
| ZR7530 | Luminal | ERBB2-amp | Positive | 1.19 | 29.86 | 8 | 156.25 | 177.00 |  |  | 8 | 17.60 | 49.80 | 16.70 |
| BT483 | Luminal | Luminal | Negative | 11.10 |  |  |  | 98.00 | 67.80 |  |  | 50 | 83.70 | 16.70 |
| MDAMB453 | Luminal | Luminal | Negative | 3.76 | 27.08 | 2.75 | 382.37 | 60.40 | 72.40 | 600.84 | 8 | 50 | 2.10 | 0.23 |
| MFM-223 | Luminal | Luminal | Negative | 1.19 | 20.20 |  | 15.19 | 9.52 | 56.80 | 356.88 |  |  | 0.91 |  |
| CAMA1 | Luminal | Luminal | Positive |  | 8.55 | 8 | 162.79 |  |  | 60.07 | 8 | 50 | 12.70 | 16.70 |
| HCC1428 | Luminal | Luminal | Positive | 1.01 |  |  |  | 97.50 | 48.00 |  |  | 50 | 12.00 | 16.70 |
| HCC1500 | Luminal | Luminal | Positive | 3.94 |  |  |  | 9.84 | 32.60 |  |  |  | 2.96 |  |
| MCF7 | Luminal | Luminal | Positive | 0.74 | 3.34 | 8 | 164.59 | 41.90 | 42.00 | 264.39 | 8 | 50 | 10.80 | 16.70 |
| MDAMB134VI | Luminal | Luminal | Positive |  |  |  |  |  |  |  |  | 0.63 |  | 0.00 |
| MDAMB175VII | Luminal | Luminal | Positive |  |  | 3.98 |  |  |  |  | 8 | 2.56 | 0.46 |  |
| MDAMB415 | Luminal | Luminal | Positive | 0.58 |  | 8 |  | 7.88 | 139.00 |  | 8 | 10.90 | 1.63 | 0.65 |
| T47D | Luminal | Luminal | Positive | 8.03 | 20.79 | 8 | 115.18 | 55.50 | 83.60 | 195.67 | 8 | 50 | 7.29 | 16.70 |
| ZR751 | Luminal | Luminal | Positive |  |  | 8 |  |  | 240.00 | 140.26 | 8 |  |  |  |
| EFM-19 | Luminal | Luminal | Positive |  | 3.86 | 8 | 0.01 |  |  | 25.35 | 8 |  | 11.50 |  |

1. Yang W, Soares J, Greninger P, Edelman EJ, Lightfoot H, Forbes S, Bindal N, Beare D, Smith JA, Thompson IR, Ramaswamy S, Futreal PA, Haber DA, et al. Genomics of Drug Sensitivity in Cancer (GDSC): a resource for therapeutic biomarker discovery in cancer cells. Nucleic Acids Res. 2013; 41: D955-61. doi: 10.1093/nar/gks1111.

2. Garnett MJ, Edelman EJ, Heidorn SJ, Greenman CD, Dastur A, Lau KW, Greninger P, Thompson IR, Luo X, Soares J, Liu Q, Iorio F, Surdez D, et al. Systematic identification of genomic markers of drug sensitivity in cancer cells. Nature. 2012; 483: 570-5. doi: 10.1038/nature11005.

3. Barretina J, Caponigro G, Stransky N, Venkatesan K, Margolin AA, Kim S, Wilson CJ, Lehar J, Kryukov GV, Sonkin D, Reddy A, Liu M, Murray L, et al. The Cancer Cell Line Encyclopedia enables predictive modelling of anticancer drug sensitivity. Nature. 2012; 483: 603-7. doi: 10.1038/nature11003.

4. Daemen A, Griffith OL, Heiser LM, Wang NJ, Enache OM, Sanborn Z, Pepin F, Durinck S, Korkola JE, Griffith M, Hur JS, Huh N, Chung J, et al. Modeling precision treatment of breast cancer. Genome Biol. 2013; 14: R110. doi: 10.1186/gb-2013-14-10-r110.
